# Conversion from a failed proximal femoral nail anti-rotation to a cemented or uncemented total hip arthroplasty device: a retrospective review of 198 hips with previous intertrochanteric femur fractures

**DOI:** 10.1186/s12891-020-03806-0

**Published:** 2020-11-30

**Authors:** Weiguang Yu, Xiulan Han, Wenli Chen, Shuai Mao, Mingdong Zhao, Xinchao Zhang, Guowei Han, Junxing Ye, Meiji Chen, Jintao Zhuang

**Affiliations:** 1 Department of Orthopaedics, The First Affiliated Hospital, Sun Yat-sen University, No. 58, Zhongshan 2nd Road, Yuexiu District, Guangzhou, 510080 China; 2 Department of Rehabilitation, The First Affiliated Hospital, Sun Yat-sen University, No. 58, Zhongshan 2nd Road, Yuexiu District, Guangzhou, 510080 China; 3 Department of Neurosurgery, The First Affiliated Hospital, Sun Yat-sen University, No. 58, Zhongshan 2nd Road, Yuexiu District, Guangzhou, 510080 China; 4 Department of Hepatobiliary Surgery, The First Affiliated Hospital, Sun Yat-sen University, No. 58, Zhongshan 2nd Road, Yuexiu District, Guangzhou, 510080 China; 5 Department of Orthopaedics, Jinshan Hospital, Fudan University, Longhang Road No. 1508, Jinshan District, Shanghai, 201508 China; 6 Department of Orthopaedics, The Affiliated Hospital of Jiangnan University, No. 1000, Hefeng Road, Wuxi, 214000 Jiangsu China; 7 Department of Pediatrics, The First Affiliated Hospital, Sun Yat-sen University, No. 58, Zhongshan 2nd Road, Yuexiu District, Guangzhou, 510080 China; 8 Department of Urology, The First Affiliated Hospital, Sun Yat-sen University, No. 58, Zhongshan 2nd Road, Yuexiu District, Guangzhou, 510080 China

**Keywords:** Failure, Revision, Complication, Outcome, Total hip arthroplasty. Cemented

## Abstract

**Background:**

At present, it is unclear which device (uncemented or cemented total hip arthroplasty [UTA or CTA, respectively]) is more suitable for the conversion of a failed proximal femoral nail anti-rotation (PFNA). The aim of this review was to assess the outcomes of failed PFNAs converted to a UTA or CTA device in elderly individuals with intertrochanteric femoral fractures (IFFs).

**Methods:**

Two hundred fifty-eight elderly individuals (258 hips) with IFFs who underwent a conversion to a UTA or CTA device following failed PFNAs during 2007–2017 were retrospectively identified from the China Southern Medical Centre (CSMC) database. The primary endpoint was the Harris Hip Score (HHS); secondary endpoint was the key orthopaedic complication rate.

**Results:**

The median follow-up was 65 months (60–69 months). Significant distinctions were observed (87.26 ± 16.62 for UTA vs. 89.32 ± 16.08 for CTA, *p* = 0.021; 86.61 ± 12.24 for symptomatic UTA vs. 88.68 ± 13.30 for symptomatic CTA, *p* = 0.026). A significant difference in the overall key orthopaedic complication rate was detected (40.8% [40/98] vs. 19.0% [19/100], *p* = 0.001). Apparent distinctions were detected in terms of the rate of revision, loosening, and periprosthetic fracture (11.2% for UTA vs 3.0% for CTA, *p* = 0.025; 13.2% for UTA vs 5.0% for CTA, *p* = 0.043; 10.2% for UTA vs 3.0% for CTA, *p* = 0.041, respectively).

**Conclusion:**

For elderly individuals with IFFs who suffered a failed PFNA, CTA devices may have a noteworthy advantage in regard to the revision rate and the rate of key orthopaedic complications compared with UTA devices, and CTA revision should be performed as soon as possible, regardless of whether these individuals have symptoms.

## Background

Despite the improvements in clinical outcomes achieved by the introduction of proximal femoral nail anti-rotations (PFNAs) for intertrochanteric femoral fractures (IFFs), a small number of elderly individuals will succumb to their disease [1]. Available treatment options involving conversion to total hip arthroplasty (THA) for a failed PFNA have been reported [2, 3], which provide the basis for further treatment strategies. A failed PFNA converted to an uncemented or a cemented THA (UTA or CTA, respectively) device has been considered a recognized option [4]. At present, which device (UTA or CTA) is more beneficial in this conversion is still controversial [4, 5]. For individuals in the 50–70 age range, a previous study [4] involving 72 conversions of failed PFNAs to a CTA device (cement; Stryker, Mahwah, NJ) exhibited that dual-cemented CTA devices had satisfactory functional outcomes, with a total complication rate of 20.8% and periprosthetic fracture rate of 4.2%. For individuals in the 40–80 age range, data from the Norwegian Arthroplasty Register [6] indicated that dual-uncemented UTA devices had an elevated risk of failure (RR 1.4; CI 1.2–1.6), which was primarily attributed to a growing risk of periprosthetic fracture (RR 5.2; CI 3.2–8.5) and dislocation (RR 2.2; CI 1.5–3.0) when compared with dual -cemented CTA devices. For individuals in the 40–50 age range, a retrospective study [7] consisted of 168 CTA devices with a mean follow-up of 10 (2–19) years, and it showed an improved clinical functional outcome, with a revision rate of 17% and a survival of 88% (95% CI: 82–94) after ten years. However, for elderly individuals aged ≥60 years old with a failed PFNA, there is no literature on the failed PFNA converted to a UTA or CTA device following previous IFFs.

Considering the limited literature on this conversion of a failed PFNA to a UTA or CTA device, we used the China Southern Medical Centre (CSMC) database to execute a retrospective review to assess clinical outcomes of this conversion of a failed PFNA to either a dual-uncemented UTA or a dual-cemented CTA device without antibiotics in the elderly population with primary IFFs.

## Methods

### Study population

Consecutive elderly individuals with IFFs who had experienced a conversion from a failed PFNA to a dual -uncemented UTA device or a dual -cemented CTA device from March 1, 2007 to March 31, 2017 were identified from the CSMC database and retrospectively analysed. The revision procedure was executed by four highly experienced orthopaedists according to previous descriptions [3, 4]. Postoperative functional rehabilitation instructions and medication interventions were based on our published literature [4]. Inclusion criteria were as follows: individuals aged ≥60 years old at the time of the conversion intervention; individuals with a primary IFF (type AO/OTA 31. A) who underwent the conversion of a failed PFNA (Smith & Nephew, Memphis, Tennessee, USA) to either a dual -uncemented UTA device or a dual -cemented CTA device without antibiotics (endoprosthetic components are shown in Table 1) as a result of a cut-out, non-union, or intolerable hip pain. Major exclusion criteria were as follows: incomplete information (i.e., the year of primary PFNA and conversion, age, sex, diagnosis, indication for revision); unidentified type of prosthesis or hybrid prosthesis; polytrauma; bilateral IFFs that may have affected the validity of the results; osteosynthesis or hip dysfunction prior to fractures; a concomitant diagnosis of inflammatory arthropathy (i.e., tuberculous arthritis or rheumatoid arthritis); diseases that affect bone metabolism (i.e., rickets, Fanconi’s syndrome or hyperparathyroidism), organ failure (i.e., chronic renal failure, heart failure), steroid dependence or resistance diseases, serious infectious diseases (i.e., acute respiratory distress syndrome), tumours, and mental disorders. Each individual had to undergo similar standardized rehabilitation instructions after index revision. Related physiotherapy (weigh-bearing and range-of-motion exercises as tolerated) was initiated immediately after revision.

**Table 1:** Manufacturer details of prostheses employed in the review

No.	Stem	Cup
UTA		
98	Corail^a^,	Reflection uncemented^b^,
CTA		
100	Exeter^c^,	Exeter^c^

^a^DePuy, ^b^Smith & Nephew, ^c^Stryker. UTA: uncemented total hip arthroplasty; CTA: cemented total hip arthroplasty

### Outcomes and variables

Follow-up occurred at 1, 6, and 12 months after revision and annually thereafter. The primary endpoint measure was functional outcome as evaluated using the HHS. Pre- and post-revision HHSs were documented. The functional results of symptomatic and asymptomatic patients prior to revision were compared separately. The secondary endpoint measures included the overall rate of key orthopaedic complications and the rate of revision, aseptic loosening, periprosthetic fracture, dislocation, and unbearable hip pain. We defined PFNA failure as any condition that required replacement with another prosthesis. We defined revision as when any component (involving femoral head and liner) or the whole prosthesis was extracted or exchanged for any reason [8]. Aseptic loosening was defined on the basis of prior report [2]. Symptomatic or asymptomatic were defined as patients having or not having symptoms (i.e., hip pain or dyskinesia) prior to revision, respectively.

### Statistical analysis

We used the Mann-Whitney U test to assess nonparametric data. Parametric data were compared using t-tests (age, BMI [body mass index], BMD [bone mineral density], and follow-up time). Categorical variables (sex, side, symptomatic/asymptomatic prior to revision, mechanism of injury, fracture pattern, comorbidities, reasons for conversion, interval from PFNA to revision, and key orthopaedic complications) were cross-tabulated and assessed per the chi-squared or Fisher’s exact tests. Kaplan-Meier method was used to illustrate the implant survival curves, and the log-rank test was used for comparison. Hazard ratios (HR) with 95% confidence intervals (CI) was calculated using the Cox proportional hazard regression model. Bias initiated by individual skeletal variation was avoided at the time of inclusion through the analysis of the contralateral hip. The level of statistical significance was set at *p* < 0.05. Statistical analyses were executed per SPSS 25.0 (IBM, Armonk, NY).

## Results

**Fig. 1:**
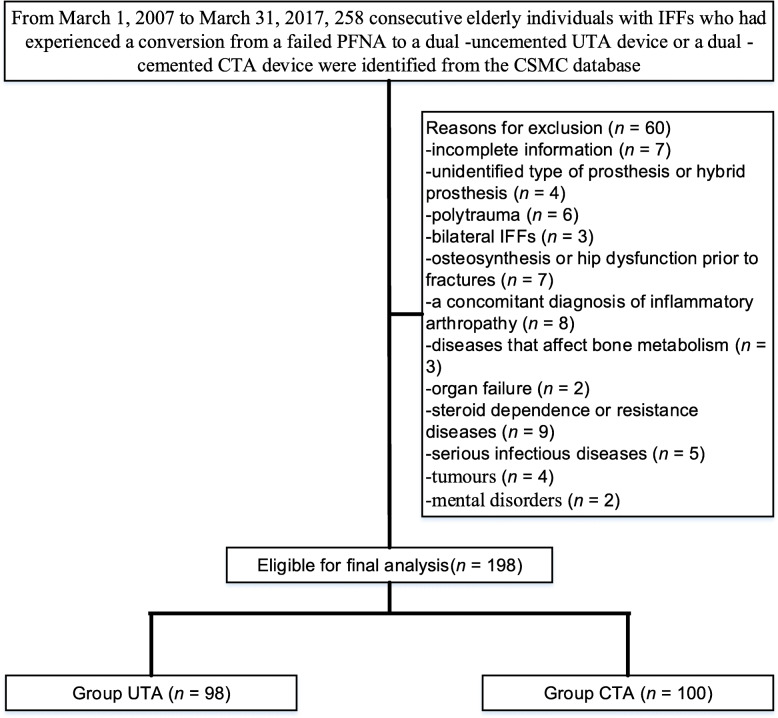
Flow diagram showing methods for identification of study population to assess the outcomes of failed PFNAs converted to a UTA or CTA device in elderly individuals with IFFs

In total, 258 patients with a failed PFNA were identified in the current study, 60 of whom did not meet the criteria for inclusion, leaving 198 patients (UTA, *n* = 98; CTA, *n* = 100) eligible for the study (Fig. 1). The mean age at the time of the revision was 66 (62–71) years for a UTA device and 66 (61–71) years for a CTA device. There were 45 and 48% males in the UTA and CTA groups, respectively. Both instability and mechanical failure were the most frequent reasons for surgery (54.1% of all revisions for UTA vs. 52.0% of all revisions for CTA). The patient characteristics were presented in Table 2. The median follow-up was 65 (60–69) months for the UTA group and 65 (61–69) months for the CTA group.

**Table 2:** Baseline data

Variable	UTA (*n* = 98)	CTA (*n* = 100)	*p*-value
Gender, M/F	45/53	48/52	0.769
Age(y)	66.84 ± 4.72	66.73 ± 5.12	0.162
BMI (kg/m^2^)	26.11 ± 5.82	26.78 ± 6.26	0.241
BMD	−3.67 ± 0.62	−3.66 ± 0.73	0.317
Side, R/L	48/50	51/49	0.776
Symptomatic/asymptomatic patients prior to revision	56/42	60/40	0.683
Mechanism of injury, No. %			0.609
Traffic	33 (33.7)	36 (36.0)	
Falling	53 (54.1)	56 (56.0)	
Direct impact	12 (12.2)	8 (8.0)	
IFFs, AO/OTA, No. %			0.629
31A1	16 (16.3)	19 (19.0)	
31A2	57 (58.2)	61 (61.0)	
31A3	25 (25.5)	20 (20.0)	
Comorbidities, No. %			0.747
Hypertension	36 (36.7)	33 (33.0)	
Diabetes mellitus	23 (23.5)	21 (21.0)	
Cerebrovascular accident	10 (10.2)	13 (13.0)	
Reasons for conversion, n%			0.954
Instability	23 (23.5)	25 (25.0)	
Mechanical failure	22 (22.4)	23 (23.0)	
Instability and mechanical failure	53 (54.1)	52 (52.0)	
Interval from PFNA to revision (y)			0.841
< 1	15 (15.3)	17 (17.0)	
1–2	53 (54.1)	56 (56.0)	
> 2	30 (30.6)	27 (27.0)	
Follow-up (mos)	65.15 ± 4.40	65.26 ± 4.21	0.103

*PFNA* Proximal femoral nail anti-rotations, *UTA* Uncemented total hip arthroplasty, *CTA* Cemented total hip arthroplasty, *HHS* Harris hip score, *ASA* American Society of Anesthesiologists, *BMI* Body mass index, *BMD* Bone mineral density

### Primary endpoint

**Fig. 2:**
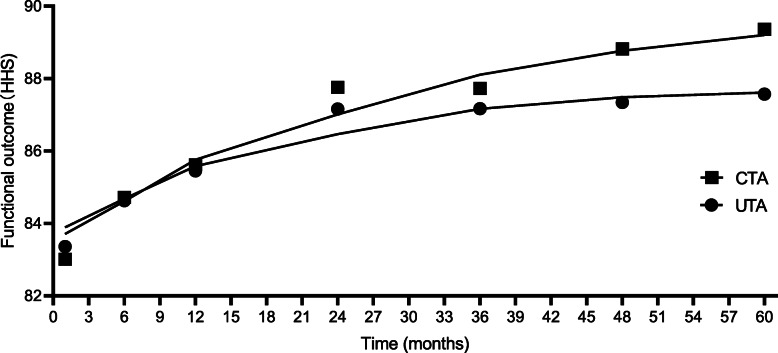
The change curve of the mean value of the two groups of functional results at each follow-up

Tables 3 and 4 show the functional results of HHSs for the two groups. Figure 2 represents the change curve of the mean value of the two groups of functional results at each follow-up. At the final follow-up, significant distinctions were observed (87.26 ± 16.62 for UTA vs. 89.32 ± 16.08 for CTA, *p* = 0.021; 86.61 ± 12.24 for symptomatic UTA vs. 88.68 ± 13.30 for symptomatic CTA, *p* = 0.026). During the first 3 years after revision, there was no distinct distinction in HHSs between groups. Starting from 3 years after revision, a higher functional score was observed in the CTA group than in the UTA group, and this advantage tend to strengthen over time. For the UTA group, significant differences were observed when comparing symptomatic and asymptomatic patients (87.26 ± 16.62 for symptomatic vs. 89.32 ± 16.08 for asymptomatic, *p* < 0.05). For the CTA group, significant differences were also noted when comparing symptomatic and asymptomatic patients (88.68 ± 13.30 for symptomatic vs. 90.54 ± 15.02 for asymptomatic, *p* < 0.05).

**Table 3:** Mid-term follow-up HHS

Month(s) postoperatively	UTA (n = 98)	CTA (n = 100)	*p*-value
1	83.36 ± 12.14	83.01 ± 12.16	0.339
6	84.63 ± 13.17	84.72 ± 13.51	0.101
12	85.45 ± 14.26	85.62 ± 14.15	0.305
24	87.16 ± 13.28	87.76 ± 13.04	0.268
36	87.17 ± 14.40	87.73 ± 12.49	0.253
48	87.34 ± 15.12	88.82 ± 15.11	0.017*
60	87.57 ± 16.02	89.36 ± 15.48	0.021*
Final follow-up	87.26 ± 16.62	89.32 ± 16.08	0.021*

*Statistically significant values

*PFNA* Proximal femoral nail anti-rotations, *UTA* Uncemented total hip arthroplasty, *CTA* Cemented total hip arthroplasty, *HHS* Harris hip score

**Table 4:** Comparison of symptomatic patients prior to revision and asymptomatic patients prior to revision

	UTA (n = 98)		CTA (n = 100)		
Month(s) postoperatively	Symptomatic (*n* = 65)	Asymptomatic (*n* = 54)	Symptomatic (n = 65)	Asymptomatic (n = 54)	*p*-value
1	81.21 ± 12.91	84.80 ± 17.43	81.19 ± 13.20	86.31 ± 14.10	0.271
6	82.49 ± 14.08	85.25 ± 16.52	82.60 ± 12.12	87.42 ± 13.49	0.104
12	84.64 ± 13.12	86.30 ± 12.97	85.32 ± 14.81	86.20 ± 11.33	0.312
24	86.33 ± 15.67	87.61 ± 16.51	87.66 ± 15.32	88.16 ± 13.72	0.203
36	86.52 ± 13.30	88.52 ± 14.85	86.84 ± 13.76	88.57 ± 17.81	0.164
48	87.87 ± 12.72	87.37 ± 15.13	88.42 ± 16.34	89.34 ± 16.26	0.029*
60	86.15 ± 14.16	88.57 ± 15.16	88.64 ± 15.33	90.16 ± 16.79	0.031*
Final follow-up	86.61 ± 12.24	88.46 ± 14.36	88.68 ± 13.30	90.54 ± 15.02	0.026*

*Statistically significant values. “*p*-value” representing the comparison of two groups of symptomatic patients; *p* < 0.05 for comparison of asymptomatic patients between groups and for comparison of asymptomatic and asymptomatic patients between groups. *PFNA* Proximal femoral nail anti-rotations, *UTA* Uncemented total hip arthroplasty, *CTA* Cemented total hip arthroplasty

### Secondary endpoint

**Fig. 3:**
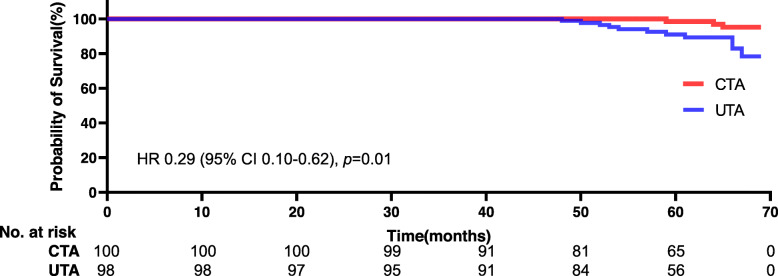
Kaplan–Meier Curves for implant revision as endpoint, unadjusted Log rank *p* = 0.01

There were 40 key orthopaedic complications in the UTA group and 19 in the CTA group. A distinct distinction in the overall key orthopaedic complication rate was detected (40.8% [40/98] vs. 19.0% [19/100], *p* = 0.001). Survival curves for implant revision showed that the 69-month unadjusted cumulative survival rates were 0.632 (95% CI, 0.614–0.657) for UTA and 0.963 (95% CI, 0.952–0.971) for CTA (HR 0.29 [95% CI 0.10–0.62], *p* = 0.01), as presented in Fig. 3. For the UTA group, 11 (11.2%) individuals underwent conversion surgery, 13 (13.2%) experienced aseptic loosening, and 10 (10.2%) had a periprosthetic fracture. For the CTA group, 3 (3.0%) individuals underwent conversion surgery, 5 (5.0%) experienced aseptic loosening, and 3 (3.0%) had a periprosthetic fracture, as presented in Table 5. During the first 3 years of follow-up, statistically significant differences failed to be detected between groups in terms of revision, aseptic loosening, or periprosthetic fracture. At the final follow-up, the rate of revision was 11.2% for UTA and 3.0% for CTA (*p* = 0.025); significant differences in the rates of the two orthopaedic complications were detected (aseptic loosening: 13.2% for UTA vs 5.0% for CTA, *p* = 0.043; periprosthetic fracture: 10.2% for UTA vs 3.0% for CTA, *p* = 0.041). Aseptic loosening (acetabular loosening and/or femoral loosening) was the most common cause of revision (72.7%[8/11] for UTA vs. 66.7%[2/3]), as presented in Table 6.

**Table 5:** Mid-term follow-up: prosthesis-related key orthopaedic complication rate

Variable, n%	UTA (n = 98)	CTA (n = 100)	*p*-value
Revision	11 (11.2)	3 (3.0)	0.025*
Aseptic loosening	13 (13.2)	5 (5.0)	0.043*
Periprosthetic fracture	10 (10.2)	3 (3.0)	0.041*
Dislocation	3 (3.1)	3 (3.0)	0.980
Unbearable hip pain	3 (3.1)	4 (4.0)	0.721

*Statistically significant values

*UTA* Uncemented total hip arthroplasty, *CTA* Cemented total hip arthroplasty

**Table 6:** Details of reasons for revision

Reason for Revision	UTA (*n* = 11)	CTA (*n* = 3)
Acetabular loosening	4	2
Femoral loosening	3	0
Acetabular and femoral loosening	1	0
Dislocation	1	1
Wear	1	0
Fracture	1	0

*UTA* Uncemented total hip arthroplasty, *CTA* Cemented total hip arthroplasty

## Discussion

This retrospective study showed that for elderly individuals who suffered PFNA failure, compared with a UTA device, a CTA device may have a noteworthy advantage in regard to the revision rate and the rate of key orthopaedic complications. Our findings are in line with those of previous studies [9–11] that a CTA device is more effective than a UTA device in primary PFNA revision. In addition, for elderly patients with clear failure of PFNAs, CTA revision should be performed as soon as possible, regardless of whether these individuals have symptoms. With the increasing numbers of failed PFNAs, expanded application of UTA or CTA devices is predictable [12]. Nonetheless, individuals are not eager to suffer from the excessive complications initiated by UTA or CTA revision for treating failed PFNAs. Thus, when a revision procedure is proposed, CTA revision for failed PFNAs could be an enticing option.

Findings from previous studies [13, 14] have demonstrated the superiority of CTA devices over UTA devices. Although significant differences in functional scores were detected, we did not detect noteworthy distinctions in the incidence of key orthopaedic complications during the first 3 years. For patients with PFNA failure, the mid-term prognosis of UTA or CTA devices remains controversial [15]. A growing body of evidence [16, 17] suggests that the differences in therapeutic efficacy between a UTA device and a CTA device for a failed PFNA were primarily in the revision rate. Nonetheless, a short-term follow-up study [18] involving 112 patients with failed PFNAs showed that there was no noteworthy distinction between a UTA device and a CTA device regarding the revision rate, and the use of UTA device did not lead to an increase in orthopaedic-related complications.

The evidence on which the decision to use a prosthesis to revise a failed PFNA was based was vague and controversial [19]. In addition, the operating specifications for reducing or avoiding mechanical complications were rarely mentioned in the previous literature [7, 20]. Consistent with previous randomized trials [14, 16], we did not observe a remarkable difference in mechanical complications between groups at the end of the 3-year follow-up. In the current study, the incidence of major orthopaedic complications was acceptable. However, there may be some differences in the comparison of quantitative variables, mainly due to the previous studies [5, 12] involving diverse research subjects, such as multi-ethnic groups, different age groups or groups with younger subjects. According to previous experience [21], UTA devices tend to be used in younger, non-osteoporotic patients, while CTA devices are frequently used in older osteoporosis patients. Based on this conclusion, we could conclude that CTA devices tend to be adopted for PFNA failure in elderly individuals. However, for younger groups, the choice of prosthesis is still controversial [13]. Recent studies [19, 22] have shown that CTA devices have noteworthy advantages in terms of revision rate and orthopaedic complications for failed PFNAs in young patients compared to UTA devices.

UTA devices are now infrequently utilised in elderly patients with osteoporosis [4]. This infrequent use is largely attributable to a regrettably high orthopaedic complication rate [2], although improvements in crosslinked polyethylene have increased the biomaterial benefits of UTA devices over CTA devices [5]. Nonetheless, the number of individuals suffering from UTA-related orthopaedic complications continues to increase. No consensus has been reached on the indications and timing of revision for PFNAs, and the rates of the revision of PFNAs differ among studies [4, 14]. Furthermore, a series of studies [23, 24] have reported that conservative management has successfully treated a large number of patients with failed PFNAs. In this situation, it is quite difficult for surgeons to manage such patients with failed PFNAs, and conservative management or revision has become an intractable alternative. However, recent evidence [2, 22] suggests that UTA or CTA revision is generally associated with a significant improvement in functional outcomes. Therefore, such intervention should be performed regardless of the complications initiated by UTA or CTA revision.

For both the UTA and CTA groups, symptomatic patients experienced greater functional improvement after undergoing revision than asymptomatic patients. However, for asymptomatic patients, a CTA device provided a significant functional improvement compared to a UTA device at the final follow-up, as evidenced by the key orthopaedic complications, but for symptomatic patients, no significant differences were detected in the functional outcomes at the final follow-up. This observation may indicate the benefits of patients undergoing CTA revision before symptoms appear. However, despite the high rate of complications, symptomatic patients could obtain more functional benefits from revision than asymptomatic patients. Notably, the functional scores of asymptomatic patients after revision did not show a significant decline, and these patients continued to maintain a high functional level. The delay of revision surgery for asymptomatic patients until after symptoms appear may lead to poor functional outcomes. However, a definitive surgical approach may be tricky to determine for some patients; therefore, we advocate a comprehensive evaluation of patients.

The present review has several limitations. First, this study was retrospective in design and, therefore, susceptible to inherent inclusion and exclusion biases that cannot be adjusted. Second, the conclusions may have been influenced by the relatively small population, inappropriate control of confounding factors, and short-term follow-up. Third, when key orthopaedic complications are used as an endpoint of the study, some complications are inevitably ignored, which may have a certain impact on the judgement of the results. However, several complications included in our study have been frequently reported in previous studies. Fourth, in the baseline data, we did not mention the patient’s occupational type or other potential medical diseases, but these potential risk factors that can lead to UTA or CTA revision may play a critical role in some patients. Finally, simple utilisation of HHSs and major orthopaedic complications as study endpoints to measure patient functional outcomes and orthopaedic complications may have limitations.

## Conclusions

The aim of the current review was to offer feasible descriptive evidence that a CTA device may be superior to a UTA device in terms of the revision rate and the rate of key orthopaedic complications in elderly patients with failed PFNAs who underwent UTA or CTA revision. For such patients with clear failure of PFNAs, CTA revision could be the preferred option, regardless of whether these individuals have symptoms prior to revision. The current findings may help resolve the ongoing debate over revision in such individuals.

## Data Availability

The data utilized are accessible from the corresponding author upon reasonable request.

## References

[1] Yu W, Zhang X, Zhu X, Yu Z, Xu Y, Zha G (2016). Proximal femoral nails anti-rotation versus dynamic hip screws for treatment of stable intertrochanteric femur fractures: an outcome analyses with a minimum 4 years of follow-up. BMC Musculoskelet Disord..

[2] Tyson Y, Rolfson O, Karrholm J, Hailer NP, Mohaddes M (2019). Uncemented or cemented revision stems? Analysis of 2,296 first-time hip revision arthroplasties performed due to aseptic loosening, reported to the Swedish hip Arthroplasty register. Acta Orthop.

[3] Smabrekke A, Espehaug B, Havelin LI, Furnes O (2004). Operating time and survival of primary total hip replacements - an analysis of 31745 primary cemented and uncemented total hip replacements from local hospitals reported to the Norwegian Arthroplasty register 1987-2001. Acta Orthop Scand.

[4] Zeng X, Zhan K, Zhang L, Zeng D, Yu W, Zhang X, et al. Conversion to total hip arthroplasty after failed proximal femoral nail antirotations or dynamic hip screw fixations for stable intertrochanteric femur fractures: a retrospective study with a minimum follow-up of 3 years. BMC Musculoskelet Disord. 2017;18. 10.1186/s12891-017-1415-6 PMC5264307 28122548

[5] Makela KT, Matilainen M, Pulkkinen P, Fenstad AM, Havelin L, Engesaeter L, et al. Failure rate of cemented and uncemented total hip replacements: register study of combined Nordic database of four nations. BMJ-Bri Med J. 2014;348. 10.1136/bmj.f7592 24418635

[6] Dale H, Borsheim S, Kristensen TB, Fenstad AM, Gjertsen JE, Hallan G (2020). Fixation, sex, and age: highest risk of revision for uncemented stems in elderly women - data from 66,995 primary total hip arthroplasties in the Norwegian Arthroplasty register. Acta Orthop.

[7] de Kam DCJ, Gardeniers JWM, Veth RPH, Schreurs BW (2010). Good results with cemented total hip arthroplasty in patients between 40 and 50 years of age. Acta Orthop.

[8] Zeng X, Zhan K, Zhang L, Zeng D, Yu W, Zhang X, et al. The impact of high total cholesterol and high low-density lipoprotein on avascular necrosis of the femoral head in low-energy femoral neck fractures. J Orthop Surg Res. 2017;12. 10.1186/s13018-017-0532-0 PMC5316144 28212664

[9] Berend ME, Smith A, Meding JB, Ritter MA, Lynch T, Davis K (2006). Long-term outcome and risk factors of proximal femoral fracture in uncemented and cemented total hip arthroplasty in 2551 hips. J Arthroplasty.

[10] Parker MI, Pryor G, Gurusamy K (2010). Cemented versus uncemented hemiarthroplasty for intracapsular hip fractures a randomised controlled trial in 400 patients. J Bone Joint Surg-Brit Vol.

[11] Liu T, Hua X, Yu W, Lin J, Zhao M, Liu J (2019). Long-term follow-up outcomes for patients undergoing primary total hip arthroplasty with uncemented versus cemented femoral components: a retrospective observational study with a 5-year minimum follow-up. J Orthop Surg Res.

[12] Sternheim A, Abolghasemian M, Safir OA, Backstein D, Gross AE, Kuzyk PR (2013). A long-term survivorship comparison between cemented and Uncemented cups with shelf grafts in revision Total hip Arthroplasty after dysplasia. J Arthroplasty.

[13] Davis CM, Berry DJ, Harmsen WS (2003). Cemented revision of failed uncemented femoral components of total hip arthroplasty. J Bone Joint Surg Am Vol.

[14] Chammout G, Muren O, Laurencikas E, Boden H, Kelly-Pettersson P, Sjoo H (2017). More complications with uncemented than cemented femoral stems in total hip replacement for displaced femoral neck fractures in the elderly a single-blinded, randomized controlled trial with 69 patients. Acta Orthop.

[15] Junnila M, Laaksonen I, Eskelinen A, Pulkkinen P, Havelin LI, Furnes O (2016). Implant survival of the most common cemented total hip devices from the Nordic Arthroplasty register association database. Acta Orthop.

[16] Kiran M, Johnston LR, Sripada S, McLeod GG, Jariwala AC (2018). Cemented total hip replacement in patients under 55 years: good results in 104 hips followed up for >= 22 years. Acta Orthop.

[17] Sandiford NA, Jameson SS, Wilson MJ, Hubble MJW, Timperley AJ, Howell JR (2017). Cement-in-cement femoral component revision in the multiply revised total hip arthroplasty results with a minimum follow-up of five years. Bone Joint J.

[18] Figved W, Opland V, Frihagen F, Jervidalo T, Madsen JE, Nordsletten L (2009). Cemented versus Uncemented Hemiarthroplasty for displaced femoral neck fractures. Clin Orthop Relat Res.

[19] Stigbrand H, Gustafsson O, Ullmark G (2018). A 2-to 16-year clinical follow-up of revision Total hip Arthroplasty using a new Acetabular implant combined with impacted bone allografts and a cemented cup. J Arthroplasty.

[20] Salemyr M, Muren O, Ahl T, Boden H, Eisler T, Stark A (2015). Lower periprosthetic bone loss and good fixation of an ultra-short stem compared to a conventional stem in uncemented total hip arthroplasty a randomized clinical trial with DXA and RSA in 51 patients. Acta Orthop.

[21] Wilson MJ, Whitehouse SL, Howell JR, Hubble MJW, Timperley AJ, Gie GA (2013). The results of Acetabular impaction grafting in 129 primary cemented Total hip Arthroplasties. J Arthroplasty.

[22] Hanly RJ, Whitehouse SL, Lorimer MF, de Steiger RN, Timperley AJ, Crawford RW (2019). The outcome of cemented Acetabular components in Total hip Arthroplasty for osteoarthritis defines a proficiency threshold: results of 22,956 cases from the Australian Orthopaedic Association National Joint Replacement Registry. J Arthroplasty.

[23] Launonen AP, Lepola V, Flinkkila T, Laitinen M, Paavola M, Malmivaara A (2015). Treatment of proximal humerus fractures in the elderly a systematic review of 409 patients. Acta Orthop.

[24] Makela KT, Matilainen M, Pulkkinen P, Fenstad AM, Havelin LI, Engesaeter L (2014). Countrywise results of total hip replacement an analysis of 438,733 hips based on the Nordic Arthroplasty register association database. Acta Orthop.

